# Functional Characterization of a Venom Protein Calreticulin in the Ectoparasitoid *Pachycrepoideus vindemiae*

**DOI:** 10.3390/insects11010029

**Published:** 2019-12-31

**Authors:** Lei Yang, Beibei Wang, Liming Qiu, Bin Wan, Yi Yang, Mingming Liu, Fang Wang, Qi Fang, David W. Stanley, Gongyin Ye

**Affiliations:** 1 State Key Laboratory of Rice Biology & Ministry of Agriculture Key Lab of Molecular Biology of Crop Pathogens and Insects, Institute of Insect Sciences, Zhejiang University, Hangzhou 310058, China; yanglei@zju.edu.cn (L.Y.); wangbei_zju@163.com (B.W.); 21816179@zju.edu.cn (L.Q.); wan-bin1234@hotmail.com (B.W.); yylqy@zju.edu.cn (Y.Y.); liumingmingzju@163.com (M.L.); wangf121@163.com (F.W.); fangqi@zju.edu.cn (Q.F.); 2 USDA Agricultural Research Service, Biological Control of Insects Research Laboratory, Columbia, MO 65203, USA; stanleyd@missouri.edu

**Keywords:** venom proteins, parasitoids, *Pachycrepoideus vindemiae*, *Drosophila*, immunity

## Abstract

Venom proteins act in the immunological interactions between parasitoids and their host insects. The effect of venom proteins on host immunity is not fully understood in pupal parasitoids. We identified the functions of a venom protein, calreticulin (PvCRT), in the pupal ectoparasitoid *Pachycrepoideus vindemiae*. Here, we report that PvCRT features a signal peptide and two conserved “calreticulin” domains. Multiple sequence alignments show that PvCRT shares 83.54% amino acid identity with CRT from both *Pteromalus puparum* and *Nasonia vitripennis*, which infers a close relationship among these three species. Using qPCR analysis, we found a lower expression level of PvCRT (0.27-fold) in the venom apparatus compared to the corresponding carcass. Immunohistochemical localization revealed that PvCRT was ubiquitously expressed in venom gland. The expression of the PvCRT gene in *Drosophila* transgenic lines via the UAS/Gal4 binary expression system reduced the self-encapsulation phenotype of *tu(1)Sz^1^* mutants. Additionally, studies on humoral immunity indicate that PvCRT does not affect the antimicrobial immune responses of the host. This work on an ectoparasitoid will increase our understanding of venom–mediated host-parasitoid interactions.

## 1. Introduction

Parasitoids are a valuable group of natural enemies used to manage arthropod pests. In the typical case, female wasps lay eggs either inside (endoparasitoid) or on the surface (ectoparasitoid) of their hosts where their larvae develop [1]. Ultimately, the host dies during the late stage of parasitoid development. The variations in the developmental strategies of endoparasitoids and ectoparasitoids suggest that parasitoids manipulate the physiology of their hosts in different ways [2]. Parasitic factors, including venom proteins [3,4], polydnaviruses (PDVs) [5], virus-like particles (VLPs) [6] and ovarian secretions [7,8] are injected into the host while the eggs are laid. Depending on the specific host/parasitoid relationship, some or all of these factors operate to manipulate host development and immunity, which are necessary for successful parasitism.

Venom proteins suppress host immunity, regulate host physiological process and change host metabolism and behavior [1,3]. Venoms are rich in bioactive proteins and peptides [1]. Despite the functional variations in the venom cocktails of endoparasitoids and ectoparasitoids, several conserved proteins appear in the venom profiles of wasps, including calreticulin (CRT). CRT is a calcium (Ca^2+^)-binding protein with multiple regulatory functions in cellular physiology. It was first identified in the endoplasmic reticulum of rabbit skeletal muscle cells [9]. CRT is a critical virulence factor in endo- and ectoparasitoid venoms [10,11,12,13,14,15,16,17]. Although they are highly conserved in amino acid sequences, a distinct set of functions occurs in different host–parasitoid models. For example, in *Cotesia rubecula* and *P. puparum*, one function of the venom CRT is to attenuate cell spreading and encapsulation behavior of host hemocytes [12]. CRT (PpCRT) from *P. puparum* may also decrease the transcript levels of host encapsulation-related genes [11]. Hosts envenomated by CRT gene-silenced *N. vitripennis* leads to increased melanization [10]. CRT in *Cotesia plutellae* venom inhibited hemocytic nodule formation in host hemolymph after challenge with *Escherichia coli* bacteria [15]. We infer that CRT in parasitoid venom mediates extensive attenuation of host cellular and humoral immunity.

As a versatile pupal ectoparasitoid, *P. vindemiae*, (Pteromalidae: Hymenoptera), has a wide host range [18,19,20,21,22]. Venom proteins are the only virulence factor for successful parasitism by *P. vindemiae*, and they are responsible for manipulating the host immune system [23]. *Drosophila* is an ideal host for studying the immunological interactions between *P. vindemiae* venom proteins and the host. Our previous study provided ample evidence that calreticulin (PvCRT) of the pupal ectoparasitoid *P. vindemiae* is present in venom. Here, we focus on the sequence, evolutionary status, immunolocalization and functional properties of the PvCRT.

## 2. Materials and Methods

### 2.1. Insect Rearing

*W^1118^* was used as the wild type control (WT), *tu(1)Sz^1^* [24] (stock ID: 5834) and *w^1118^, P{Cg-GAL4.A}2* (Cg) [25] (stock ID: 7011) were obtained from the Bloomington stock center. All *Drosophila* lines were raised on standard corn meal medium (1 L water, 105 g corn flour, 75 g brown sugar, 7.5 g agar, 6.25 mL propionic acid and 20 g yeast extract) at 25 °C with 60 ± 5% relative humidity and a 16L:8D photoperiod. The *P. vindemiae* colony was kindly provided by Prof. Yongyue Lu (South China Agricultural University, Guangzhou, China) in January 2016. *P. vindemiae* were bred by parasitizing the pupae of WT at 25 °C with a 14L:10D photoperiod as described in [26]. After eclosion, adults were held in glass containers and maintained on 10% (v/v) honey solution.

### 2.2. Sequence and Phylogenetic Analysis

The coding DNA sequence of PvCRT (GenBank: MN583584) was obtained by searching the venom apparatus transcriptome database. The SignalP-5.0 server and the simple modular architecture research tool (SMART) were used for predicating the signal peptide and conserved domains, respectively [27,28]. A schematic diagram of the amino acid sequence structure was drawn with software IBS 1.0.1 [29]. The protein tertiary structure was modeled by the homology-modeling server SWISS-MODEL, as described in [30,31]. We conducted multiple sequence alignments based on the deduced amino acid sequences using Clustal Omega [32]. Alignment results were visualized using ENDscript 3.0 [33]. The phylogenetic tree was constructed based on the maximum likelihood method using Mega 6 software with 1000 bootstrap values, and further edited and visualized using the Interactive Tree of Life (iTOL) v3 [34].

### 2.3. Venom Apparatus Collection and Isolation of Total RNA

Mated female wasps aged 2–7 days were chilled at 4 °C for 10 min and then rinsed in sterile phosphate-buffered saline (PBS, pH 7.2) followed by dissection in PBS with 1 unit/μL RNase inhibitor (Vazyme, Nanjing, China) on an ice plate under a Leica MZ 16A stereomicroscope (Leica, Wetzlar, Germany). The venom apparatus and carcass (minus the venom apparatus) were collected in 1 mL of TRIzol reagent (Invitrogen, Carlsbad, CA, USA). Total RNA was extracted as per the manufacturer’s protocol. The quantity of the total RNA samples was determined by NanoDrop 2000 (Thermo Scientific, Wilmington, DE, USA) and stored at −80 °C for subsequent experiments.

### 2.4. cDNA Synthesis and Quantitative Real-Time PCR

The first-strand complementary DNA (cDNA) was synthesized from total RNA using PrimeScript™ RT Reagent Kit with gDNA Eraser (Takara, Beijing, China). qPCR was carried out using the ChamQ^TM^ SYBR^®^ qPCR Master Mix (Vazyme, Nanjing, China) and run on a CFX96™ Real-Time PCR Detection System (Bio-Rad, Hercules, CA, USA) following the manufacturer’s instructions. The specific qPCR primers were designed using AlleleID 6 software (PREMIER Biosoft, Palo Alto, CA, USA) (Supplementary Materials, Figure S1), gene expression levels were normalized to the reference gene (28S rRNA) [35]. The qPCR programs were set as following: enzyme activation at 95 °C for 30 s, followed by 40 cycles with denaturation at 95 °C for 5 s, annealing at 60 °C for 30 s, and melting curve analysis. The mRNA expression levels were determined by the comparative 2^−^^△△CT^ method [36].

### 2.5. Gene Cloning

cDNA from *P. vindemiae* venom apparatus was used as a template to clone the *PvCRT*. The PCR reaction included 5 μL cDNA template (20 ng/μL), 1.5 μL of each gene-specific primer (10 μmol/L), 25 μL KOD One^TM^ PCR Master Mix (Toyobo, Shanghai, China) and 17 μL sterile water. PCR primers were designed using the Primer Premier 6 software (PREMIER Biosoft International, Palo Alto, CA, USA) (Supplementary Materials, Figure S1). The reaction conditions were 35 cycles of 98 °C for 10 s, 60 °C for 5 s, and 68 °C for 7 s. The pUASTattB plasmid (GenBank: EF362409.1), a cloning vector for producing *Drosophila* transgenic lines, was digested by EcoRI and KpnI (Thermo, Carlsbad, CA, USA). PCR amplification product was analyzed on 1.0% agarose gel, and recombined into the enzyme-digested vector using ClonExpress Ultra One Step Cloning Kit (Vazyme, Nanjing, China). Finally, positive cloning was verified by DNA sequencing.

### 2.6. Gal4-Driven Expression of PvCRT

Transgenic lines were generated by the *Drosophila* Resources and Technology Platform (Shanghai Institute of Life Sciences, Chinese Academy of Sciences). Appropriate crosses were performed to obtain a homozygous line carrying two copies of PvCRT (UAS-PvCRT). To explore the functions of PvCRT on host immunity, Gal4-driven expression in immune tissues (fat body and hemocytes) was initiated by crossing the *Cg-Gal4* (Cg) lines to UAS-PvCRT lines and the offspring was denoted Cg > UAS-PvCRT [37]. The hybrids between Cg lines and WT lines (Cg/WT), and the crossed offspring between WT lines and UAS-PvCRT lines (WT/UAS-PvCRT) were used as control. All crosses were performed at 25 °C. We established a positive control of encapsulation in pupal *Drosophila* using the temperature-sensitive mutant *tu(1)Sz^1^*. At 28 °C *tu(1)Sz^1^* larvae form melanotic tumors, involving the encapsulation of abnormal caudal fat body regions [24]. In vivo self-encapsulation experiments were conducted by crossing virgin temperature-sensitive mutant *tu(1)Sz^1^* to Cg > UAS-PvCRT lines. Cg/WT lines were used as control. Once crossed, offspring were maintained at 28 °C for further self-encapsulation observation. Larvae and pupae were then scored for the *tu(1)Sz^1^* phenotype as described [38].

### 2.7. Genomic DNA Extraction and PCR Detection

Sets of ten *D. melanogaster* pupae were collected into 1.5 mL centrifuge tubes followed by genomic DNA extraction according to the manufacture’s protocol using Fastpure Cell/Tissue DNA Isolation Mini Kit (Vazyme, Nanjing, China). Total RNA of *Drosophila* pupae was extracted and cDNA was synthesized as described. PCR amplification was conducted using PvCRT gene-specific primers (Supplementary Materials, Figure S1). PCR reactions were performed as described and amplification products were analyzed on a 1.0% agarose gel, cloned into TA/Blunt-Zero Vector (Vazyme, Nanjing, China) and then sequenced.

### 2.8. Western Blotting

Venom proteins from *P. vindemiae* were collected as described [39], and total proteins of the carcass, whole body of *P. vindemiae* or *D. melanogaster* were separately extracted using Minute™ total protein extraction kit for animal cultured cells/tissues (Invent Biotechnologies, Beijing, China). The protein concentration was determined by a modified Bradford protein assay Kit (Sangon Biotech, Shanghai, China). Protein extracts (1 μg) were denatured in sodium dodecyl sulfate polyacrylamide gel electrophoresis (SDS-PAGE) sample-loading buffer and separated on 12% SDS-PAGE gels, then transferred onto polyvinylidene fluoride membranes (Merck Millipore, Darmstadt, Germany). A 5% non-fat milk solution was used for blocking the membranes and the primary antibody was incubated at 4 °C overnight. Rabbit polyclonal antibodies against PvCRT, β-actin were diluted 1:2000 and 1:4000, respectively. Goat anti-rabbit IgG-horseradish peroxidases conjugate diluted 1:4000 (GenScript, Nanjing, China) was used as the secondary antibody for 1 h incubation. The signal was developed using the super signal west dura extended duration substrate (Thermo Scientific, Waltham, MA, USA) and detected with a UVP ChemiDoc-It Imaging System (Upland, UVP Company, CA, USA).

### 2.9. Immunofluorescence Staining

Immunofluorescence staining was conducted as described previously [40]. Briefly, the venom apparatus of 4-day old female wasps was dissected and fixed with 4% polyformaldehyde for 30 min, followed by rinsing with 0.3% phosphate buffered saline with Triton-X 100 solution (PBST) three times. Subsequently, samples were blocked for 1 h with 5% goat serum (Sangon Biotech, Shanghai, China), diluted in PBST and washed for 20 min with PBST three times. The rabbit anti-PvCRT primary antibody (diluted 1:100 in 5% goat serum) was incubated for 48 h at 4 °C. Rabbit serum that had not been immunized was used as control. Samples were washed for 20 min with PBST three times and incubated with the Dylight 488-conjugated goat anti-rabbit secondary antibody diluted 1:200 (Abbkine, Redlands, CA, USA) for 48 h at 4 °C. We mounted the samples using SlowFade™ Gold Antifade Mountant with DAPI (Life Technologies, Carlsbad, CA, USA). The immunofluorescence images were recorded under a Zeiss LSM 810 confocal microscope (Carl Zeiss SAS, Jena, Germany) and clarified by Zeiss LSM ZEN 2010 software (Carl Zeiss SAS, Jena, Germany).

### 2.10. Bacterial Infection and Survival Analysis of D. melanogaster Adults

We inoculated 1 mL LB medium with *Pseudomonas aeruginosa* or *Staphylococcus aureus*, and allowed them to grow at 37 °C until OD_600 nm_ reached 0.03 and 0.1, respectively. We centrifuged 1 mL of each culture for 3 min at 5000× *g* and discarded the supernatant. The bacterial pellets were washed 3× with 1 mL PBS, re-suspended in 1 mL PBS and stored at 4 °C for inoculation. We collected 5–7 day old male flies and anesthetized them with CO_2_. A thin metal needle was sterilized with ethanol before dipping into the bacterial solution and the lateral side of the fly’s thorax was pricked. We transferred the pricked flies with a brush to a sterile vial containing corn-meal fly medium and incubated them at 25 °C. Flies were collected at indicated time points. Uninfected flies were used as controls. Total RNA extraction and qPCR were performed as described above. Primers for *diptericin* (*Dpt*) and *drosomycin* (*Drs*) genes were used to quantify the expression levels of antimicrobial peptides, ribosomal protein L32 (*RpL32*) was used as internal control [41]. The survival graphs represent one representative experiment out of three independent repeats with at least 20 flies per genotype.

### 2.11. Data Analysis

The gene expression levels for qPCR were analyzed by unpaired two-tailed Student’s *t* Test and one-way ANOVA followed by Tukey’s multiple comparison tests. The penetrance data were analyzed by the Fisher Exact test. All statistical analyses were carried out by the data processing system (DPS) package version 9.50 and statistical significances were marked with asterisks (ns: no significant difference, *: *p* < 0.05, **: *p* < 0.01, ***: *p* < 0.001) or different letters (*p* < 0.05) [42]. All figures were plotted using GraphPad Prism 7.0 (GraphPad, San Diego, CA, USA).

## 3. Results

### 3.1. Sequence Analysis of PvCRT

**Figure 1:**
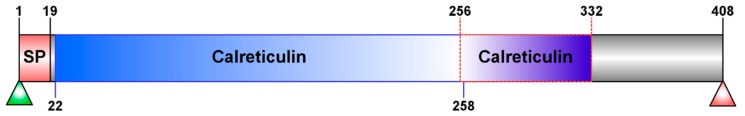
Amino acid sequence structure analysis of the calreticulin (PvCRT) in *P. vindemiae*. Blue boxes denoted two conserved “calreticulin” domains (22–258 aa and 256–332 aa). Initiation codon and termination codon were highlighted with triangles. SP: signal peptide (1–19 aa).

The coding DNA sequence (CDS) of PvCRT obtained by searching the transcriptome of venom apparatus was validated by gene cloning and sequencing. Our analysis showed the full-length CDS of the *PvCRT* gene is 1224 bps, encoding 408 amino acids with a predicted molecular weight of 47.65 kDa and an isoelectric point of 4.39. In agreement with previous reported CRTs, PvCRT features a signal peptide (1–19 aa) in the N-terminus and two conserved “calreticulin” domains (Figure 1), which may be crucial for its functional performance. However, unlike the PpCRT, [11] we did not identify the “coiled-coil” domain in PvCRT.

**Figure 2:**
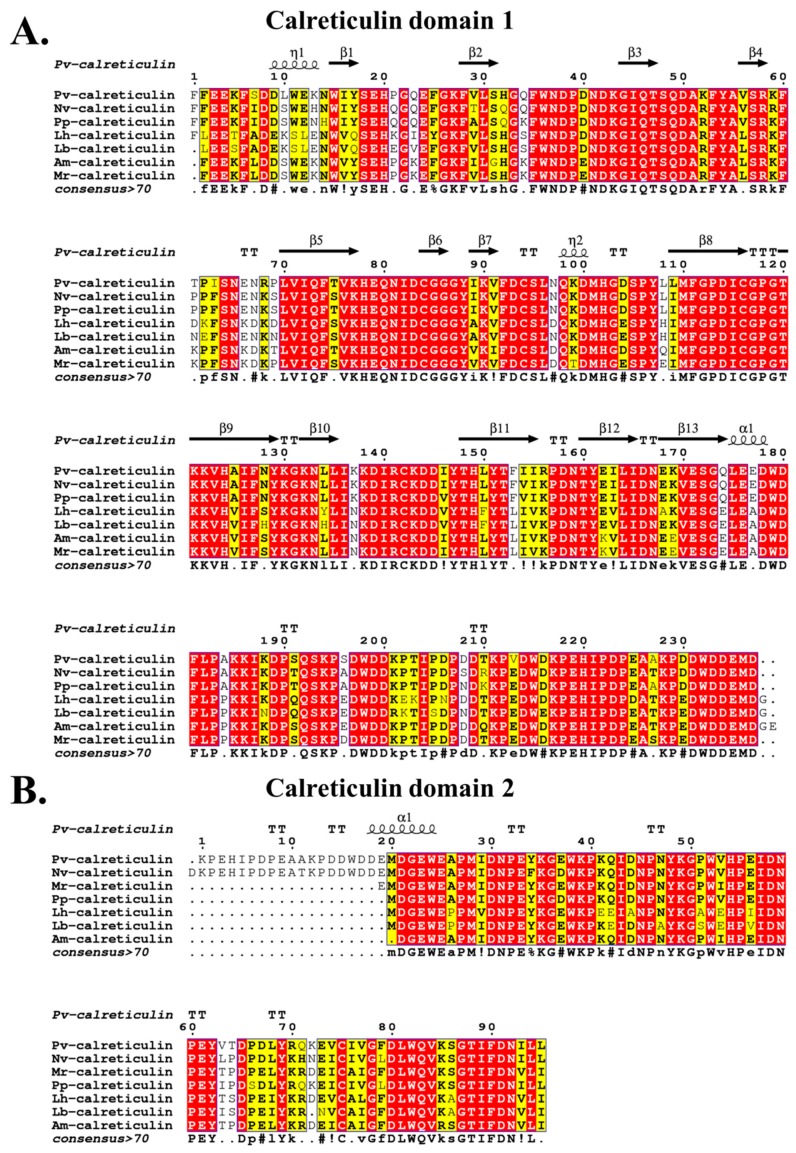
Multiple sequence alignments based on “calreticulin” domain 1 (**A**) and “calreticulin” domain 2 (**B**). Pv, *P. vindemiae*; Nv, *N. vitripennis* (NP_001155151.1); Pp, *P. puparum* (ACZ68113.1); Lh, *Leptopilina heterotoma* (comp326_c0_seq1) and Lb, *Leptopilina boulardi* (comp263_c0_seq1) [43]; Am, *Apis mellifera* (XP_006559569.1); Mr, *Megachile rotundata* (XP_003701110.1). Multiple sequence alignments were conducted by Clustal Omega and visualized by ESPript 3.0 [32,44]. The conserved amino acid residues were highlighted, red means the entirely conserved amino acid residues and yellow represents the 70–100% conservation in amino acid residues.

The PvCRT shared the highest amino acid identity (83.54%) with venom CRT from *N. vitripennis* (BlastP, E-value = 0) and *P. puparum* (BlastP, E-value = 0). Multiple sequence alignments based on the “calreticulin” domains demonstrated that PvCRT has highly conserved properties with other hymenopteran insects (Figure 2).

### 3.2. Phylogenetic Analysis

**Figure 3:**
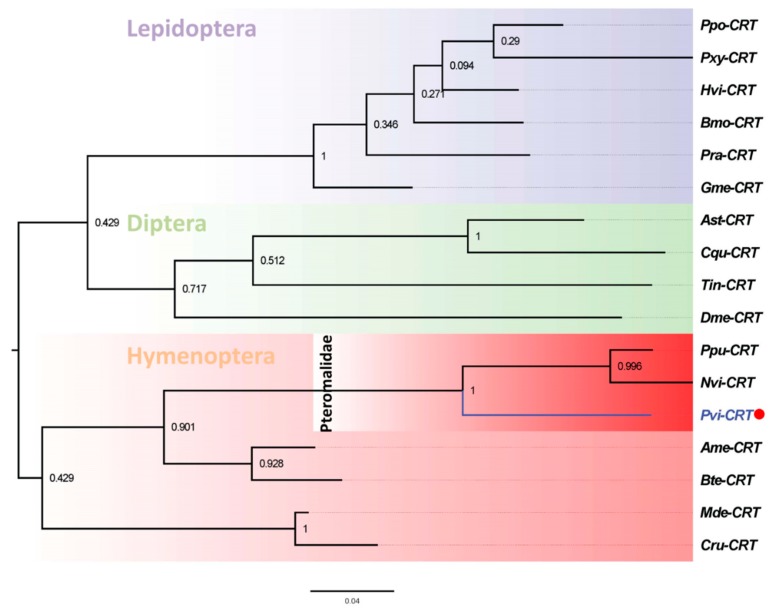
Phylogenetic tree of CRTs from *P. vindemiae* and other insects. The phylogenetic tree was constructed by the maximum likelihood method based on CRTs from *P. vindemiae* and another 17 insects using the program Mega 6 with 1000 bootstrap values. The GenBank accession numbers of 17 CRTs were listed as follows: *Pvi* (*P. vindemiae*); *Dme* (BAA85379.1, *D. melanogaster*); *Ast* (AEM05956.1, *Anopheles stephensi*); *Cqu* (XP_001848824.1, *Culex quinquefasciatus*); *Ppo* (BAM19116.1, *Papilio polytes*); *Hvi* (PCG67628.1, *Heliothis virescens*); *Bmo* (NP_001037075.1, *Bombyx mori*); *Pra* (ACJ07154.1, *Pieris rapae*); *Gme* (BAB79277.1, *Galleria mellonella*); *Pxy* (ADN06079.1, *Plutella xylostella*); *Ppu* (ACZ68113.1, *P. puparum*); *Nvi* (NP_001155151.1, *N. vitripennis*); *Ame* (XP_006559569.1, *A. mellifera*); *Bte* (XP_003403200.1, *Bombus terrestris*); *Mde* (XP_008559929.1, *Microplitis demolitor*); *Cru* (AAN73309.1, *C. rubecula*); *Tin* (APU54816.1, *Triatoma infestans*).

We chose 17 CRTs for phylogenetic reconstruction [15,45], which indicated that CRTs from hymenopteran insects clustered together and diverged from other orders. Among the clustered hymenopteran CRTs, PvCRT is more closely related to *N. vitripennis* and *P. puparum,* as seen in the sequence analyses (Figure 3).

### 3.3. Transcriptional Profiles of PvCRT

**Figure 4:**
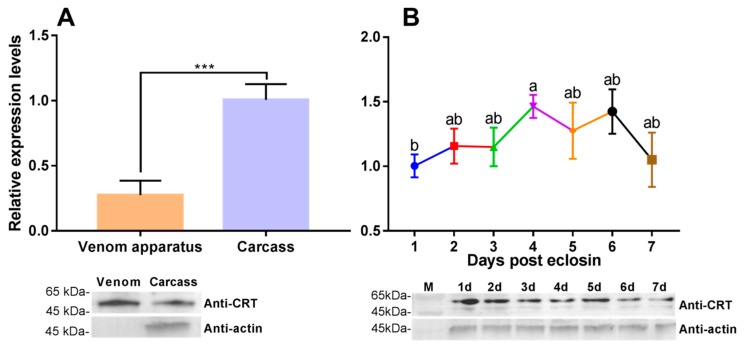
Expression of PvCRT in different tissues and developmental stages. (**A**) Protein expression profiles of PvCRT in venom apparatus (*n* ≥ 200) and carcass (*n* = 10). (**B**) Relative mRNA levels in female wasps of PvCRT at 1–7 days after emergence (*n* = 10). All values in the figure are represented as mean ± standard deviation. Significant differences are labeled above the bars (unpaired two-tailed Student’s *t* Test, ***: *p* < 0.001) or different letters (one-way ANOVA followed by Tukey’s multiple comparison test, *p* < 0.05).

The transcriptional profiles of the venom apparatus and carcass were compared (Figure 4A). We recorded lower PvCRT expression in the venom apparatus (0.27-fold) relative to the carcass. Western blotting also showed the expression of the gene in the venom apparatus and carcass. The accumulation of mRNAs encoding PvCRT over 1–7 days post-eclosion in female adults shows fairly stable expression on days 2–3 and 5–7 post-eclosion (Figure 4B). The mRNA level appeared to reach a peak on day 4, although it only differed significantly from day 1. The small numerical differences do not suggest a biological significance (Figure 4B). Immunoblotting also indicated the presence of PvCRT in the female adults over days 1–7 post-eclosion.

### 3.4. Immunohistochemical Visualization of PvCRT in Venom Apparatus

**Figure 5:**
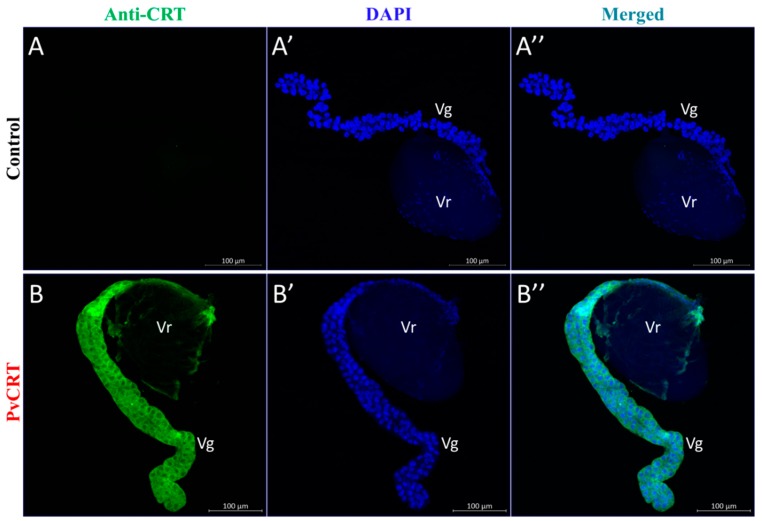
Immunolocalization of PvCRT antigen in venom apparatus, including venom gland (Vg) and venom reservoir (Vr). (**A**–**A’’**). PvCRT was detected using rabbit anti-PvCRT polyclonal antibody and Dylight 488-conjugated goat anti-rabbit secondary antibody. (**B**–**B’’**). Rabbit serum that had not been immunized was used as control. Cell nuclei were stained with DAPI (**A’**,**B’**).

Immunoblotting shows that PvCRT is expressed in the venom apparatus. We localized the PvCRT antigen within the venom apparatus, shown as green staining in the venom gland (Vg) and, with less staining in the venom reservoir (Vr) (Figure 5).

### 3.5. Construction of PvCRT Transgenic Drosophila

**Figure 6:**
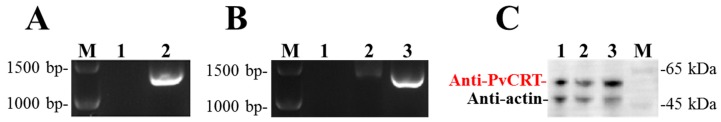
The expression of PvCRT in transgenic *Drosophila*. (**A**) The detection of PvCRT in pupal *Drosophila* by PCR. The temples of *Drosophila* genomic DNA were listed as follows: lane 1: WT, lane 2: UAS-PvCRT, M: DNA Ladder. (**B**) Validation of transcribed PvCRT in pupal *Drosophila*, cDNA temples were listed as follows: lane 1: Cg/WT, lane 2: WT/UAS-PvCRT, lane 3: Cg > UAS-PvCRT, M: DNA Ladder. (**C**) Immunoblotting in total proteins using anti-PvCRT antibody. Proteins were separately extracted from pupal *Drosophila* lines listed as follows: lane 1: Cg/WT, lane 2: WT/UAS-PvCRT, lane 3: Cg > UAS-PvCRT, M: Protein Ladder.

We produced *Drosophila* transgenic lines carrying a UAS transgene encoding the PvCRT protein (UAS-PvCRT) and confirmed the presence of PvCRT in *Drosophila* by PCR. Figure 6A shows a specific band of about 1200 bp in UAS-PvCRT line, but not in the WT. The PvCRT was validated by DNA sequencing. The expression of the PvCRT was initiated by crossing the UAS-PvCRT lines to Cg driver (Cg > UAS-PvCRT) [11,12,13,14,15,16,17,18]. We validated the transcription of PvCRT by PCR using cDNA from Cg > UAS-PvCRT, Cg/WT and WT/UAS-PvCRT lines as temples. We found a specific band was amplified in Cg > UAS-PvCRT lines and confirmed the PvCRT by DNA sequencing (Figure 6B). This shows that PvCRT was transcribed in Cg > UAS-PvCRT lines. We recorded a specific band corresponding to the predicted size in both *Drosophila* transgenic lines and controls by western blotting (Figure 6C). We speculate this is due to the high similarity in amino acid sequence (80.5%) between PvCRT and *D. melanogaster* CRT (Supplementary Figure S1), implying the high potential for cross-reactivity.

### 3.6. PvCRT Inhibits Encapsulation of the Host

**Figure 7:**
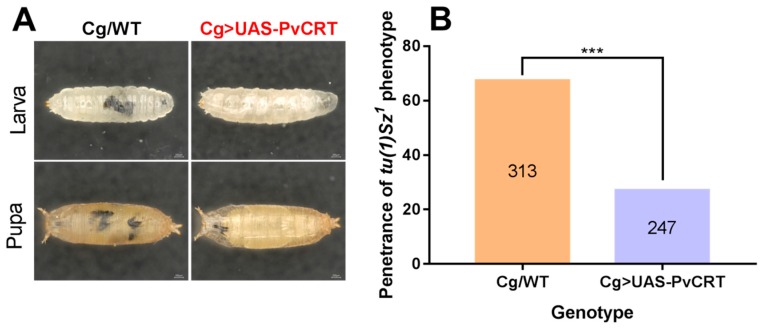
PvCRT inhibited encapsulation of the host. (**A**) Microscopic inspection of self-encapsulation phenotype in larvae and pupae between control and transgenic lines. (**B**) Penetrance of *tu(1)Sz^1^* phenotype in the pupal *Drosophila*. Significant difference was marked with “***” (*p* < 0.001, Fisher Exact test), the numbers indicated independent replicates for each treatment.

In vivo encapsulation assays showed a higher rate of self-encapsulation in larvae and pupae of the Cg/WT *Drosophila* parental lines, whereas the *tu(1)Sz^1^* phenotype was significantly rescued in Cg > UAS-PvCRT parental lines (Figure 7). We conclude that PvCRT inhibited encapsulation of the host.

### 3.7. The Roles of PvCRT in Antimicrobial Immunity of the Host

**Figure 8:**
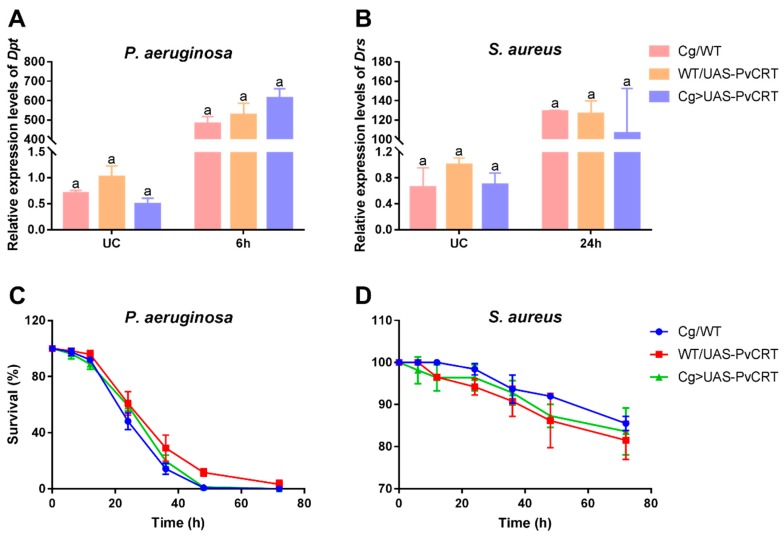
(**A**,**B**) Expression levels of antimicrobial peptides *diptericin* (*Dpt*) and *drosomycin* (*Drs*) in *Drosophila* adults after infection with *P. aeruginosa* and *S. aureus* (*n* = 5). UC: unchallenged. (**C**,**D**) *Drosophila* adults were infected with bacteria and monitored for survival (*n* ≥ 18). One-way ANOVA between different groups were performed followed by Tukey’s multiple comparison tests, and the same letters mean no significant difference.

Bacterial challenge led to multi-fold increases in mRNAs encoding *Dpt* at 6 h post-infection by gram-negative bacteria, *P. aeruginosa*, but there were no differences between transgenic flies and control (Figure 8A). Similar challenges with the gram-positive *S. aureus* led to increased mRNAs encoding *Drs* at 24 h. Again, there were no differences between transgenic lines and control (Figure 8B). We further tested the involvement of PvCRT in host survival after bacterial infection. In line with the above results, transgenic flies did not show an increased susceptibility to these infections compared to the control (Figure 8C,D). Thus, we infer that PvCRT is not involved in the antibacterial immunity of the host.

## 4. Discussion

As a cosmopolitan wasp, *P. vindemiae* attacks the puparia of many cyclorrhaphous Diptera. They successfully develop from the hosts previously parasitized by other parasitoids, indicating that *P. vindemiae* has the potential for expanding its host range [46]. Females of *P. vindemiae* parasitize the invasive spotted wing drosophila, which provides one of the most promising biological resources to control *D. suzukii* [47]. The efficiency of biological control on spotted-wing drosophila can be improved through conservation or augmentative releases of *P. vindemiae* [48]. These explorative works illustrate that *P. vindemiae* could be developed into a biological control agent.

Female *P. vindemiae* inject venom proteins into their hosts while laying eggs. The venom proteins act in host immune regulation [23]. In general, venom proteins act in inhibiting immunity, interrupting development, and regulating the metabolism of parasitoid hosts [4]. Parasitoids evolve venom proteins to suppress host immune reactions such as hemocyte spreading, encapsulation and melanization reactions [3]. This basic principle drives the research on functional studies of venom proteins, such as Vn50, Vn1.5, GAP, sarco/endoplasmic reticulum calcium ATPase (SERCA), CRT, Serpin, α-amylase [3,10,12,13,15,38,49,50,51,52,53,54,55,56]. CRT has been characterized in many wasps, including *C. rubecula*, *Microctonus* sp., *P. puparum*, *C. plutellae*, *N. vitripennis*, *L. boulardi*, *L. heterotoma* and so on [11,13,14,15,43,57]. Our study adds new information on CRT *P. vindemiae* venom.

CRTs suppress host cellular and humoral immunity [10,11,12]. In the *C. rubecula*/*P. rapae* model, venom CRT inhibits hemocyte spreading and encapsulation of their host in a dose-dependent manner [12]. Similarly, PpCRT suppressed the cellular immunity of *P. rapae* hemocytes [11]. In the endoparasitoid *C. plutellae*, CRT inhibited nodule formation in its host *P. xylostella* [15]. Siebert et al. reported that CRT from *N. vitripennis* venom inhibits melanization of its host *Sarcophaga bullata* [10]. Analysis of the calculated PvCRT amino acid sequence shows a putative signal peptide of 22 amino acids in the N-terminal region and two highly conserved “calreticulin” domains analogous to other parasitoid CRTs. The coiled-coil motif does not occur in PvCRT. The coiled-coil domain from PpCRT is responsible for its entry into host hemocytes [11]. On the basis of multiple sequence alignments, PvCRT features higher homology with CRTs from *N. vitripennis* and *P. puparum*, from which we infer similar protein functions. Phylogenetic analysis indicates PvCRT clustered with *P. puparum* and *N. vitripennis*. These proteins may have evolved from the same ancestor. More distant relationships indicate that PvCRT diverged from other insect orders, implying their functional differences.

CRT is a protein with multiple cellular functions in invertebrates and vertebrates [9]. PvCRT expression occurred in the carcass and venom, as seen in other parasitoids [10,11,12], indicating it probably acts in host and parasitoid. Despite their lower abundance, PvCRT may act in inhibiting host immune response, as reported for *PpS1V* in *P. puparum* [55]. The expression pattern of PvCRT both in venom and non-venom apparatus imply that a novel functionalization of PvCRT occurred during the co-evolution between *P. vindemiae* and its hosts. We thus suspect that it was recruited into venoms to perform venom functions [58], and the questionnaire would have been more useful if it had asked participants about the origin of PvCRT.

The *Drosophila* transgenic lines integrated PvCRT, which was transcribed and expressed by the Cg driver. Crossing the Cg/WT *Drosophila* lines with *tu(1)Sz^1^* mutants led to encapsulation of their own tissue in a manner similar to wasp egg encapsulation [38]. The self-encapsulation phenotype of *tu(1)Sz^1^* was significantly rescued in *Drosophila* transgenic lines in contrast to control flies. These results agreed with the suppression of host hemocytic encapsulation observed in PpCRT [11]. We speculate that PvCRT created a calcium burst in a similar manner with the SERCA, which inhibits encapsulation [38]. Infection either with *P. aeruginosa* or *S. aureus* led to high levels of mRNAs encoding antimicrobial peptides, albeit with no differences between Cg > UAS-PvCRT and Cg/WT, WT/UAS-PvCRT *Drosophila* lines. Our additional survival experiments did not reveal a role for PvCRT in increasing susceptibility to these bacterial infections. We infer that PvCRT does not act in host antimicrobial immunity.

## 5. Conclusions

While endo- and ectoparasitoids are closely related at the phylogenetic level, their life histories and embryonic development differ. Grbic and Strand reported that embryonic development of the ectoparsitoid *Bracon hebetor* is similar to free-living insects, with relatively large, yolky eggs, while the endoparasitoid *Aphidius ervi* lays small, yolk-free eggs [59]. Hence, ectoparasitoids differ from endoparasitoids in fundamental ways. The PvCRT facilitates studies of another axis of ecdo- and endoparasitism—the influence of the parasitoids on host immunology. Our work on this axis indicates considerable differences in the biological significance of PvCRT and probably many other proteins in venoms from endo- and ectoparasitoids. Continued work along this axis will yield new, fundamental insights into parasitoid histories.

## Supplementary Materials

The following are available online at https://www.mdpi.com/2075-4450/11/1/29/s1, Figure S1: multiple sequence alignment between PvCRT and *Drosophila* CRT, Table S1: primers used in the main text.
