# Supplementary figures and images for: Functional Characterization of a Venom Protein Calreticulin in the Ectoparasitoid Pachycrepoideus vindemiae
Source: Insects. 2019 Dec 31;11(1):29. doi: 10.3390/insects11010029 (PMC7023170; doi:10.3390/insects11010029)

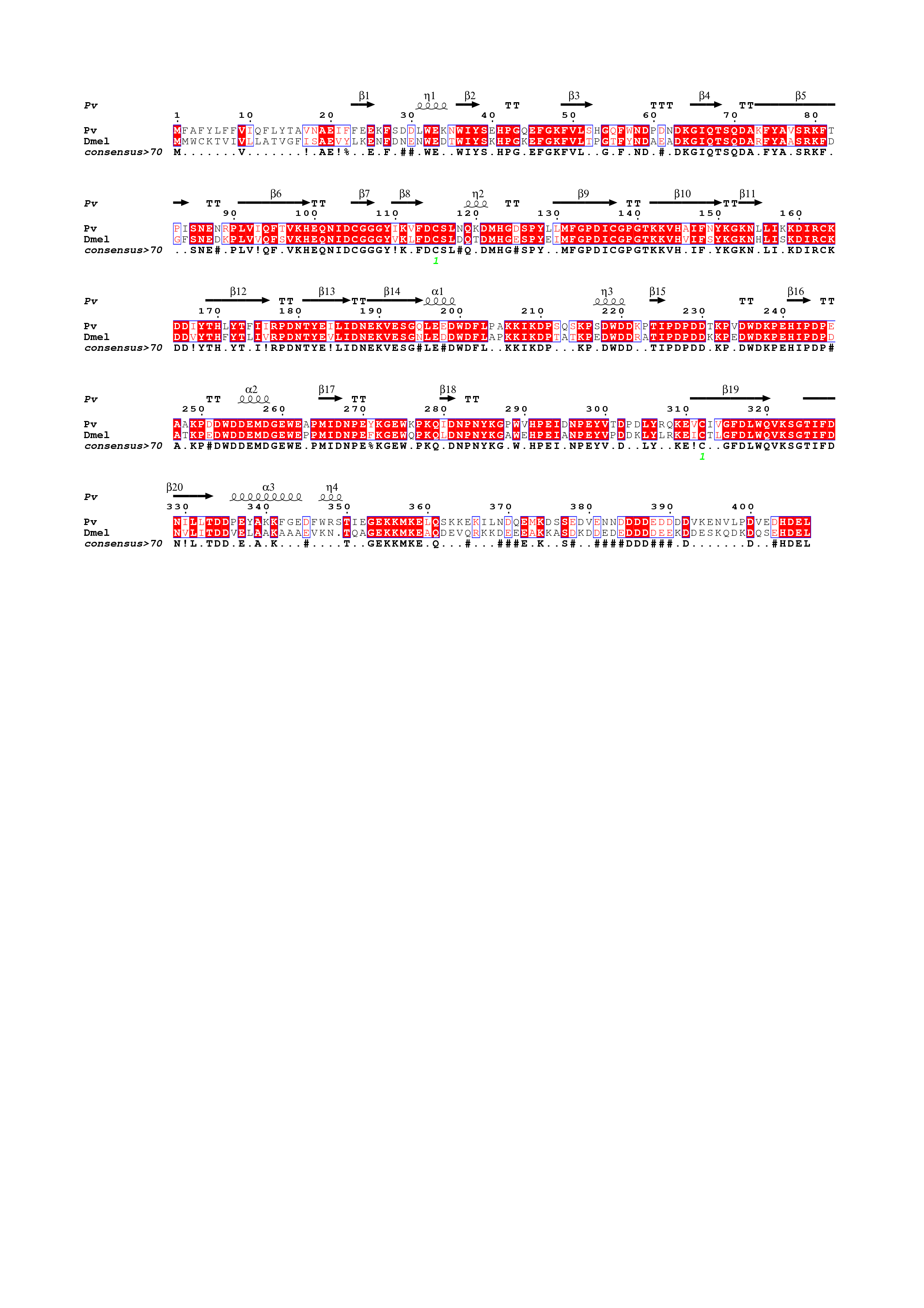

Supplement: Supplementary file 1 [file insects-11-00029-s001.zip › Supplementary material/Supplementary figure 1. Multiple sequence alignments between PvCRT and Drosophila CRT.tiff]
